# Merging evans syndrome with mucopolysaccharidosis type II: a case report

**DOI:** 10.3389/fped.2026.1784387

**Published:** 2026-05-04

**Authors:** Xinrui Wang, Jing Zhang, Yanhui Tang, Chunyan Liu, Peng Hu, You Yang, Hongying Chen

**Affiliations:** 1 Department of Pediatrics, Children Hematological Oncology and Birth Defects Laboratory, The Affiliated Hospital of Southwest Medical University, Sichuan Clinical Research Center for Birth Defects, Luzhou, Sichuan, China; 2 Department of Pediatrics Growth and Health Care, The Guangan People’s Hospital, Guangan, Sichuan, China; 3 Department of Radiology, The Affiliated Hospital of Southwest Medical University, Luzhou, Sichuan, China

**Keywords:** case report, evans syndrome (ES), hematopoietic stem cell transplantation (HSCT), iduronate-2-sulfatase (IDS), mucopolysaccharidosis type II (MPS II)

## Abstract

Mucopolysaccharidosis type II (MPS II) is an X-linked recessive lysosomal storage metabolic disorder caused by pathogenic mutations in the iduronate-2-sulfatase (IDS) gene. Herein, we report the case of a 2-year-old male patient diagnosed with concurrent Evans syndrome (ES) and MPS II, who presented with severe anemia, thrombocytopenia, recurrent respiratory tract infections, and typical clinical manifestations of MPS II. Laboratory examinations showed decreased hemoglobin (Hb) and platelet (PLT) counts, a positive direct Coombs test, presence of anti-platelet antibodies, elevated urinary glycosaminoglycan levels, and complete absence of IDS enzyme activity. Whole exome sequencing identified a novel compound heterozygous nonsense mutation in the IDS gene: c.380_383dup GCTA, p.Y128X. The patient received hematopoietic stem cell transplantation (HSCT) and underwent long-term follow-up. During the follow-up period, the patient's IDS enzyme activity returned to normal levels, and no further recurrence of ES was observed. This study reports a novel pathogenic mutation of the IDS gene, and represents the first documented case of concurrent ES and MPS II in a toddler. HSCT has been confirmed to be an effective therapeutic strategy for this extremely rare comorbid condition. Additionally, we discuss the potential pathogenic association between mucopolysaccharidosis (MPS) and ES, and present a systematic summary of the clinical management of this patient, to provide a reference for improved identification and treatment of similar clinical cases in the future.

## Introduction

Mucopolysaccharidosis (MPS) is a rare lysosomal storage disorder characterized by multi-system involvement, a progressive clinical course, and insidious early symptoms. It is primarily caused by the deficiency of specific lysosomal hydrolases, which induces progressive accumulation of glycosaminoglycans (GAGs) across multiple tissues (1).

To date, MPS has been categorized into 7 distinct subtypes, of which type II MPS (MPS II) was first identified by Canadian physician Charles Hunter in 1917 (2). MPS II is an X-linked recessive multi-system disorder caused by pathogenic mutations in the iduronate-2-sulfatase (IDS) gene. Functional deficiency of the IDS enzyme blocks the degradation of dermatan sulfate and heparan sulfate, leading to their deposition in various tissues and organs (3). The clinical manifestations of MPS II are heterogeneous, ranging from severe skeletal deformities, airway obstruction, and cardiomyopathy to progressive neurological decline. These manifestations adversely impact patient growth and development, as well as respiratory, cardiac, and neurological function, resulting in a substantial reduction in patients' quality of life (4, 5). The present case report describes a male pediatric patient diagnosed with MPS II who also presented with Evans syndrome (ES). This co-occurrence of the two conditions has not been reported previously. In this patient, we identified a novel compound heterozygous nonsense mutation in the IDS gene, which expands the known mutational spectrum of IDS associated with MPS II, and implies a potential pathogenetic association between MPS II and hematological disorders. Furthermore, this case demonstrates the successful application of hematopoietic stem cell transplantation (HSCT) in the simultaneous treatment of both MPS II and ES, providing valuable clinical evidence and guidance for the clinical management of similar cases.

## Case presentation

A one-year-old patient was admitted to hospital with skin petechiae, fever, seizures, and bicytopenia. On admission, the patient's vital signs were as follows: temperature 38.5 °C, heart rate 136 beats per minute, respiratory rate 41 breaths per minute, and blood pressure 125/74 mmHg. Physical examination revealed pale mucous membranes, scattered slate-gray macules over the back and buttocks, pinpoint hemorrhages on the trunk and lower extremities, and a 4-cm mature surgical scar in the right groin. Cardiac examination was unremarkable, while pulmonary auscultation detected coarse breath sounds accompanied by fine crackles. Abdominal distension was observed; the liver was palpable approximately 3 cm below the right costal margin and 1 cm below the xiphoid process, and the spleen was palpable approximately 2 cm below the left costal margin. According to the patient's parents, the patient was born full-term via elective cesarean section, with no history of birth trauma or perinatal hypoxia. No family history of similar hematological disorders was reported. A gray pigmented nevus was noted on the patient's back at birth, which gradually faded over time. The patient developed recurrent respiratory tract infections after birth, and underwent inguinal hernia repair at 4 and 8 months of age. Since infancy, the patient has been complicated by severe anemia, growth and developmental delay, and moderate malnutrition. Laboratory tests showed severe thrombocytopenia (18 × 10^9/L) and decreased hemoglobin (Hb) level (28 g/L), accompanied by elevated reticulocyte count (13.32%) and increased liver enzyme levels (ALT 84.9 U/L, AST 166.6 U/L). Platelet-associated antibody testing was positive, and both direct and indirect Coombs tests were strongly positive. Bone marrow aspirate analysis demonstrated hypercellular hematopoiesis (Supplementary Figure S1). Imaging examination showed enlarged cerebral ventricles on cranial computed tomography (CT) and bilateral pneumonia on chest CT. The patient was diagnosed with ES, as he presented with concurrent onset of immune thrombocytopenic purpura (ITP) and autoimmune hemolytic anemia (AIHA). The treatment regimen included red blood cell and platelet transfusion, plasma exchange, intravenous immunoglobulin therapy, intravenous antibiotics, and corticosteroid therapy, which was subsequently switched to oral administration. After five months of oral corticosteroid maintenance therapy, the Coombs test turned negative, and the medication was discontinued (Supplementary Figure S2). However, hepatosplenomegaly was still present in the patient (Supplementary Figure S3). Ten months after treatment discontinuation, the Coombs test reverted to positive, accompanied by decreased Hb and platelet(PLT) counts.

**Figure 1:**
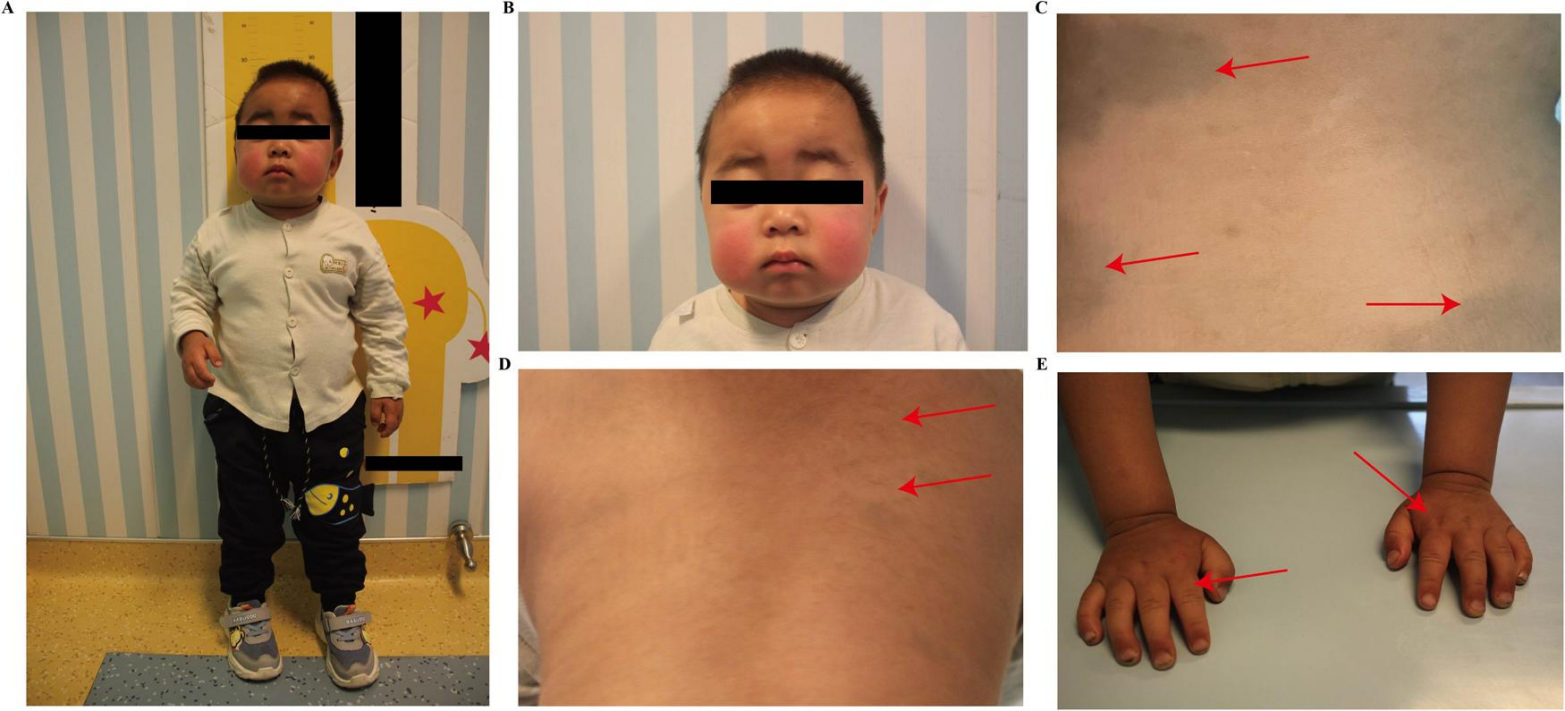
Typical clinical manifestations of the patient. **(A)** The patient's dysmorphic facial features and height characteristics; **(B)** The patient's dysmorphic facial features; **(C)** Mongolian spots on his back; **(D)** Asymptomatic ivory-white papules on his back; **(E)** Contracture of fingers.

**Figure 2:**
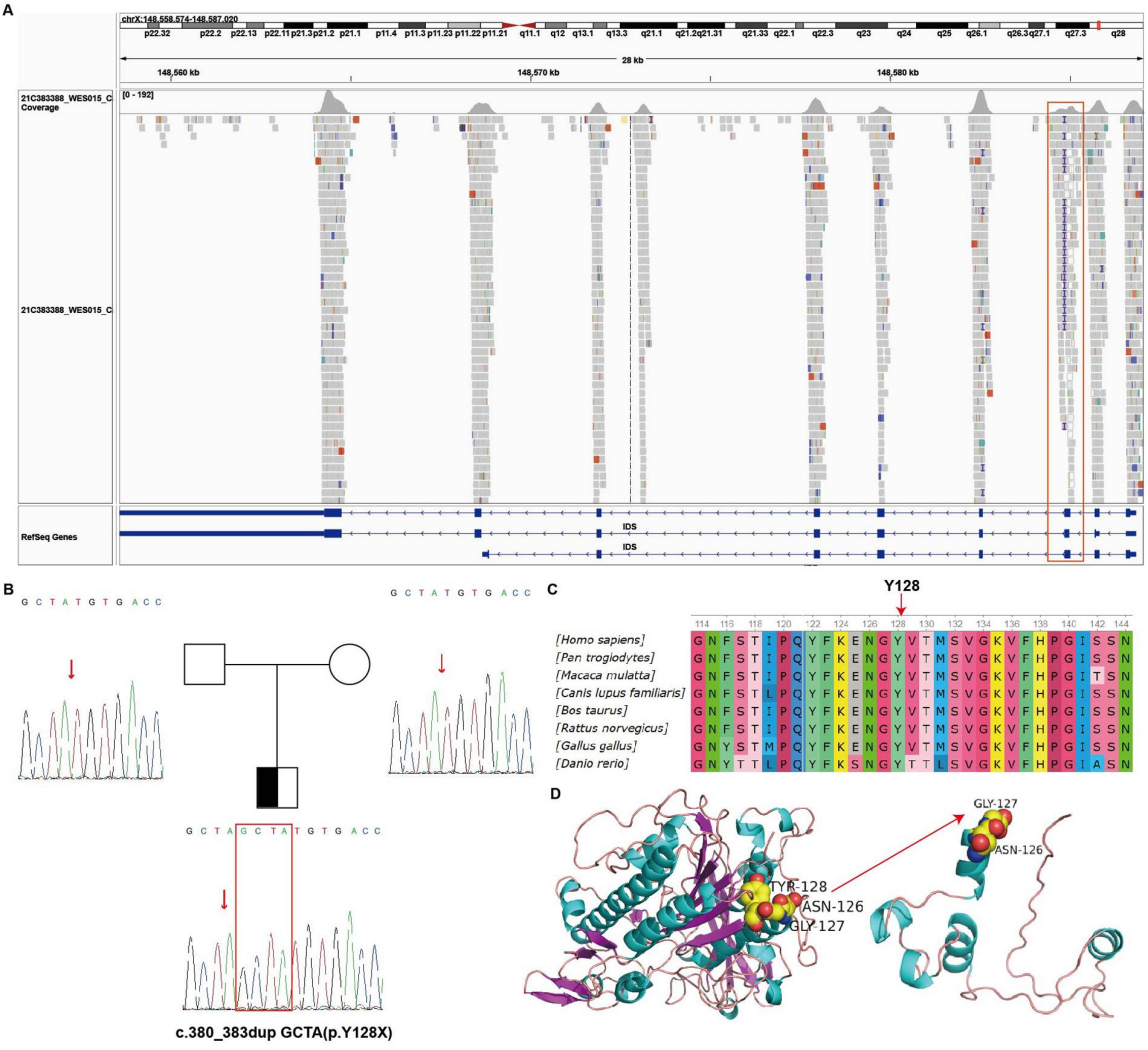
Status of IDS gene mutation. **(A)** IGV (Integrative Genomics Viewer) visualization of IDS gene; **(B)** WGS showed that the proband had a *de novo* mutation; **(C)** Amino sequence alignment indicated that the mutation site is conserved among different species; **(D)** Structural modeling revealed that Y128 located on an *α* helix, and the production of truncated protein may be pathogenic.

**Figure 3:**
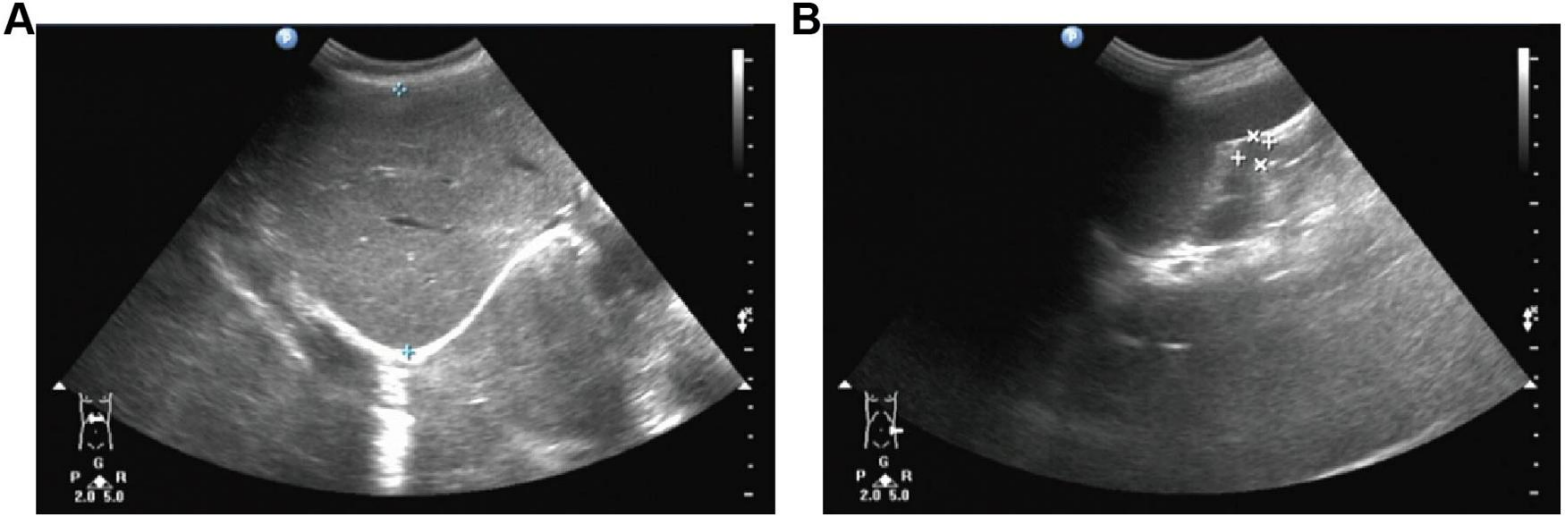
Abdominal ultrasound. **(A)** Splenomegaly; **(B)** Hepatomegaly.

**Figure 4:**
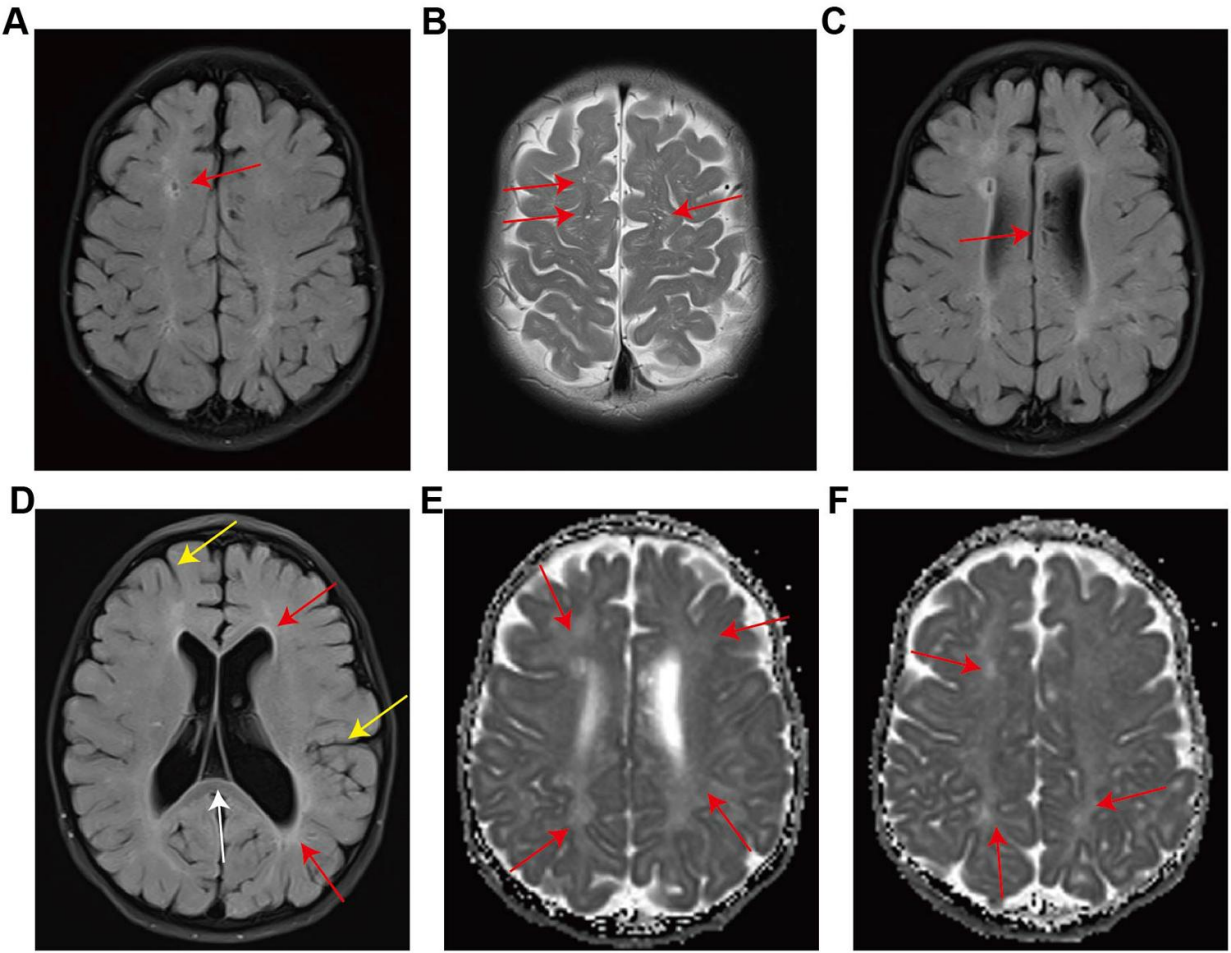
Cranial MRI. **(A,C,D)** Flair sequence image; **(B)** T2WI image; **(E,F)** Apparent diffusion coefficient sequence image.

At 2 years of age, the patient's parents reported that the child presented with nocturnal snoring, increased frequency of bowel movements, and developmental delay. Anthropometric measurements recorded a body weight of 10 kg, a body height of 85 cm (upper limb length: 53.4 cm; lower limb length: 31.6 cm), and a head circumference of 49 cm. All measurements were consistent with global growth retardation and developmental delay (Supplementary Figure S4). Physical examination revealed pale mucous membranes, coarse facial features, prominent forehead, thickened eyebrows, flattened nasal bridge, and thickened lips (Figures 1A,B). Cutaneous examination of the back identified scattered slate-gray patches along with “cobblestone"-like ivory papules (Figures 1C,D). A 4-cm chronic surgical scar was observed in the inguinal region. Digital contractures were clearly evident (Figure 1E). Enlarged lymph nodes were palpable in the cervical and inguinal regions. Cardiac examination yielded normal findings, while respiratory auscultation detected coarse breath sounds. The abdomen was distended, with a reducible umbilical hernia and detectable hepatosplenomegaly. Subsequent laboratory investigations showed a Hb level of 89 g/L, a PLT count of 139 × 10^9/L, and a reticulocyte percentage (RETIC%) of 5.81%. Both direct and indirect Coombs tests returned positive results. Pulmonary function testing demonstrated mild-to-moderate airway obstruction, and bilateral hearing screening failed to obtain valid responses. Assessment with the Gesell Developmental Scale revealed significantly delayed overall cognitive development relative to age-matched peers. Given the patient's recurrent AIHA, hepatosplenomegaly, and abnormalities in growth, development, and facial morphology, genetic testing is indicated to identify potential underlying inherited metabolic disorders. Whole exome sequencing identified a heterozygous mutation, c.380_383dupGCTA (p.Y128X), in exon 3 of the IDS gene. This nonsense mutation is a *de novo* variant (Figures 2A–C) that results in the translation of a truncated protein product (Figure 2D). Dried blood spot testing detected no residual IDS enzyme activity. The variant was not detected in either parent or the patient's three older sisters (Supplementary Figure S5). A clinical diagnosis of MPS II was established based on these findings. Subsequent testing confirmed IDS enzyme activity of 0.000 nmol/h/mL, and urinary glycosaminoglycan (GAG) analysis returned a positive result. Imaging examinations included abdominal ultrasound, which confirmed hepatosplenomegaly (Figure 3). Thoracolumbar spine radiography showed ovoid vertebral bodies with beak-like projections at the anteroinferior margins (Supplementary Figure S6A). Skeletal radiography also demonstrated small proximal phalanges, thickened distal phalanges, and paddle-shaped ribs (Supplementary Figure S6B, E). The skull was disproportionately enlarged, with increased cranial volume and hypoplasia of the facial skeleton (Supplementary Figure S6C-D). The cranial base angle was increased, consistent with a flattened cranial base (Supplementary Figure S6C). The sella turcica was flattened and presented a hook-shaped morphology (Supplementary Figure S6C). The fifth proximal phalanx of the wrist had a small distal end and a flattened proximal end, exhibiting a characteristic bullet shape (Supplementary Figure S6F). Cranial magnetic resonance imaging revealed dilated Virchow-Robin spaces in bilateral centrum ovale regions (Figures 4A,B). Diffuse white matter degeneration was observed adjacent to the bilateral lateral ventricles (Figure 4C), and the anterior and posterior horns of the lateral ventricles appeared rounded (Figure 4D, red arrow), consistent with concurrent hydrocephalus. The sixth ventricle was also visualized (Figure 4D, white arrow). Deepening and widening of the cerebral sulci were noted (Figure 4D, yellow arrow), indicative of cerebral atrophy. Focal restricted diffusion was detected around the bilateral lateral ventricles and centrum ovale regions (Figures 4E,F). Transthoracic echocardiography revealed no structural or functional abnormalities (Supplementary Figure S7). While awaiting bone marrow typing results, the patient received one month of enzyme replacement therapy. The patient's full biological sister was confirmed to be a 10/10 high-resolution human leukocyte antigen (HLA) match. Multidisciplinary consultation, with participation from departments of ophthalmology, dentistry, otolaryngology, traditional Chinese medicine, hematology-oncology, and radiology, was conducted to develop the transplantation regimen. The pre-transplant conditioning regimen consisted of cyclophosphamide (Cy), fludarabine (Flu), intravenous busulfan (IVBu), thiotepa (TT), and antithymocyte globulin (ATG). Prophylaxis against graft-versus-host disease (GVHD) was administered with a combination of cyclosporine A (CsA), mycophenolate mofetil (MMF), and methotrexate (MTX). Three days prior to the scheduled transplantation, the patient experienced a febrile seizure with a body temperature of 39.2 °C, presenting with unresponsiveness, fixed gaze, and generalized muscular rigidity. After completion of all pre-transplant preparations, peripheral blood stem cells were successfully infused from the donor sister (20 years old, blood type A+) (Supplementary Table S1). Full donor chimerism was consistently maintained in the bone marrow throughout post-transplant follow-up, with no evidence of GVHD. At follow-up, the patient's growth parameters had normalized to age-appropriate ranges, cognitive function had improved, and hepatosplenomegaly had regressed. No cases of severe anemia or febrile seizures were reported. IDS enzyme activity normalized (Supplementary Table S2), and PLT counts were consistently maintained within the normal reference range. During clinical follow-up, both direct and indirect Coombs tests converted to negative. Cranial and joint imaging examinations revealed no abnormal significant changes. Regular follow-up will be continued for the patients.

## Discussion

MPS II, also referred to as Hunter syndrome, is a rare X-linked recessive inherited disorder caused by pathogenic mutations in the IDS gene (OMIM accession: 309900) (6). The present study reports a pediatric case of MPS II caused by a novel IDS mutation, which was accompanied by ES. After identifying the pathogenic mutation and confirming no familial history of MPS II, co-segregation genetic testing was performed for the proband's family, which revealed no carriers of the identified mutation or at-risk relatives. The IDS gene is located on the long arm of the X chromosome (Xq28), consists of 9 exons and 8 introns, and encodes a 550-amino-acid polypeptide (7). IDS mutations can occur across any exon, with mutations in exons 9, 8, and 3 being the most prevalent (8). The mutation detected in this case was located in exon 3, which is consistent with findings from previous studies. To date, 729 distinct pathogenic mutations causing MPS II have been documented, including deletions, insertions, and single-nucleotide substitutions (encompassing missense, nonsense, and splice-site mutations), with missense mutations accounting for the largest proportion (9). In the reported case, whole-exome sequencing identified a previously unreported hemizygous mutation: c.380_383dupGCTA (p.Y128X) in the IDS gene. Pediatric Evans syndrome (pES) is defined as the simultaneous or sequential onset of ITP, AIHA, and/or autoimmune neutropenia (AIN) in individuals younger than 18 years old (10).

A literature review of MPS case reports retrieved from PubMed identified that MPS can affect multiple organ systems, including the nervous system, ophthalmic system, otorhinolaryngology (ENT) region, dentition, cardiovascular system, respiratory system, hematological system, immune system, urinary system, gastrointestinal system, and skeletal system (Supplementary Table S3). Among reported cases, hematological involvement was recorded in 52 cases, accounting for 6.4%, and all 52 cases included complete clinical data (Supplementary Table S4). To date, no cases of MPS II comorbid with ES have been reported in the literature.

Current treatment options for MPS II include supportive care, enzyme replacement therapy (ERT), gene therapy, HSCT, and substrate reduction therapy (SRT) (11). However, palliative supportive care cannot alter disease progression, and neither gene therapy nor SRT is currently widely available for routine clinical application (11). At present, clinical management of MPS II mainly relies on a combination of ERT and HSCT (12). A study by Vinicia Assunta Polito and Maria Pia Cosma demonstrated that systemic administration of the AAV2/5-CMV-hIDS vector in MPS II mouse pups successfully corrects central nervous system (CNS) abnormalities and reduces GAG accumulation. This therapeutic effect is achieved by enabling the IDS enzyme to cross the blood-brain barrier, rather than relying on direct infection of brain cells by the viral vector (13). Stuart Ellison et al. further verified that in a MPS II mouse model, gene therapy based on hematopoietic stem cells modified with a lentiviral vector expressing apolipoprotein E peptide (ApoEII), a blood-brain barrier targeting peptide, can produce sustained systemic therapeutic benefits for both peripheral and central nervous system pathologies, while maintaining a good safety profile (14). Existing conventional ERT is ineffective for CNS lesions because the IDS enzyme cannot cross the blood-brain barrier (14). In the present case, the patient achieved a favorable response to HSCT: platelet levels returned to the normal range, hemolytic anemia resolved, and improvements in growth, development, and cognitive function were observed. These clinical observations indicate that HSCT may exert clinical efficacy in treating the underlying metabolic disorder of MPS II and its associated complications such as ES.

C. Peters and W. Krivit previously reported a case of severe AIHA occurring after HSCT in a patient diagnosed with MPS IIB (15). In contrast, the proband in our case already had severe AIHA before HSCT, and no severe recurrence of anemia was observed after transplantation. Burak Uz et al. reported a 20-year-old male patient diagnosed with Hunter syndrome at birth who developed thrombocytopenia; based on physical examination and auxiliary examinations, the authors considered a possible diagnosis of ITP. They hypothesized that multi-system GAG accumulation combined with immune-mediated mechanisms and/or chronic antigen stimulation may lead to the development of ITP (16).

Lymphoproliferation, which is defined by lymphadenopathy, splenomegaly, hepatomegaly, or lymphocytic organ and tissue infiltration, can be the presenting sign of rare disorders, including inborn errors of immunity [IEI; e.g., autoimmune lymphoproliferative syndrome (ALPS) and related disorders] and inborn errors of metabolism [IEM; e.g., Gaucher disease(GD)] (17). ALPS is a hereditary immune disorder caused by mutations in genes involved in the FAS signaling pathway, characterized by defective FAS-mediated apoptosis (18, 19). ALPS is clinically defined by the manifestation of lymphoproliferation and autoimmune cytopenias (20). Other disorders presenting these hallmark features are generally categorized as autoimmune lymphoproliferative immunodeficiencies (ALPID) (20). ALPID, a phenotypic subgroup within the spectrum of IEI, can present with breakdown of immune homeostasis and immune dysregulation (21). GD is a canonical example of a lysosomal storage disorder, categorized as a rare autosomal recessive genetic disease caused by pathogenic mutations in the GBA1 gene (17). Notably, Maurizio Miano et al. analyzed a set of ALPS-associated immunological indicators in GD patients, including double-negative T cells (DNTs), B220⁺ DNTs, CD27⁺ cells, T-reg/HLA-DR ratio, as well as circulating levels of IL-10, IL-18 and vitamin B12 (22). Their study confirmed that patients with GD can present with ALPS-like features and FAS-mediated apoptosis defects, indicating that screening for GD should be incorporated into the diagnostic workflow for patients presenting with an ALPS-like phenotype (22). Notably, the ALPID phenotype may apply to some children currently diagnosed with ES (20). A separate study by Alix E. Seif et al. found that a substantial proportion (47%) of pediatric patients diagnosed with ES had an underlying etiology consistent with ALPS (23). Synthesizing the available evidence, an ALPS-like phenotype can be detected in IEM, which complicates the clinical diagnosis of patients presenting with concurrent ES. Given that both MPS and GD are classified as lysosomal storage disorders, we hypothesize that the pathogenesis of MPS complicated by ES may similarly involve immune dysregulation and signaling pathway abnormalities homologous to those reported in GD. Nevertheless, this hypothesis requires further empirical investigation to verify its validity. Immune system abnormalities have also been documented in other lysosomal storage disorders, including Niemann-Pick Disease (NPD) and Fabry Disease (FD). Existing studies show that early microglial activation and astrocyte proliferation occur in both mouse models and patients with Niemann-Pick disease type C (NPC), and this inflammatory response acts as a key pathological mechanism driving neurodegenerative progression (24). Further work has demonstrated that in Npc1-deficient mice, loss of functional NPC1 protein amplifies STING signaling via inhibition of lysosomal degradation pathways; combined with the upstream priming effect, this ultimately leads to severe neurological impairment (25). Consistently, knockout of either Sting1 or Irf3 (but not Cgas) significantly reduces microglial activation, mitigates Purkinje neuron loss in the cerebellum, and improves motor function (25). In FD, abnormal accumulation of globotriaosylceramide (Gb3) plays a central role in triggering excessive activation of the complement system. This results in a marked elevation of complement components including C1qC, C3, C4 and CFB, as well as their active fragments C3a and C5a, in the bodily fluids and tissues of both FD patients and corresponding animal models (26).

Dogan et al. proposed that enzyme replacement therapy may induce thrombocytopenia in patients with MPS VI, also referred to as Maroteaux-Lamy syndrome (27). The patient in the present study received one month of enzyme replacement therapy prior to HSCT, which may have contributed to the development of thrombocytopenia, yet cannot account for the onset of severe AIHA. Inusha Panigrahi et al. also observed that thrombocytopenia can be correlated with Epstein–Barr virus (EBV) infection (28). The team put forward that EBV infection may serve as a potential trigger for chronic thrombocytopenia accompanied by acute exacerbation and bleeding-induced anemia, especially among Asian patient cohorts; nevertheless, no evidence of EBV infection was detected in our patient. Glycosylation is a fundamental process in biological and biochemical recognition, mediating a broad spectrum of events ranging from fertilization and embryonic development to pathological states including infection, allergy, inflammation, and cancer (29). Of all glycosylation subtypes, N-glycosylation is a highly conserved glycan modification that modulates the function of more than 7,000 human proteins, and plays a critical role in protein folding, intracellular trafficking, and signal transduction. Accordingly, glycan modifications are indispensable to a wide array of physiological and pathological processes, and exert profound effects on multiple dimensions of biological function (30). Yuchen Pan et al. developed a novel method for complete *α*-selective glycosylation, and verified the performance of fluorogenic substrates—specifically 4-methylumbelliferyl glycosides—for activity analysis of N-sulfoglucosamine sulfohydrolase (SGSH) and *α*-N-acetylglucosaminidase (NAGLU). The authors further employed these substrates to quantify SGSH and NAGLU activity in tissue samples from MPS IIIA and IIIB model mice (31). In MPS, deficiency of specific enzymes disrupts GAG metabolism, which in turn perturbs normal cellular glycosylation processes. This perturbation can result in either abnormal accumulation of glycosylation products or impaired synthesis thereof. Furthermore, excessive accumulation of GAGs may remodel the composition and structure of the extracellular matrix, consequently altering the glycosylation profile of proteins that interact with cells. Such alterations can disrupt multiple physiological functions, ultimately driving the multi-organ and multi-tissue dysfunction typically observed in MPS patients. This cascade of biochemical and cellular perturbations underscores the complex interplay between enzyme deficiency and the overall pathophysiology of MPS. In addition, our patient had a history of febrile seizures and no family history of similar clinical manifestations. Cranial magnetic resonance imaging (MRI) identified diffuse white matter degeneration, hydrocephalus, and cortical atrophy. On this basis, we hypothesize that the patient's seizures are likely associated with these intracranial pathological changes.

## Conclusion

The co-occurrence of MPS II with ES is clinically rare, and no relevant case reports have been published to date. For patients presenting with hematological manifestations including anemia and thrombocytopenia alongside characteristic clinical features, it is critical to consider the possibility of rare disorders such as MPS after completing routine evaluations for hematological diseases. Accordingly, comprehensive auxiliary examinations including genetic testing should be performed to achieve a definitive diagnosis. The present case indicates a potential association between MPS II and autoimmune diseases. We hypothesize that the underlying pathogenic mechanisms may involve abnormal immune system activation, as well as the deleterious effect of lysosomal dysfunction on the hematological system. Furthermore, the favorable outcome of HSCT in this case demonstrates that HSCT may be an effective therapeutic option for patients with MPS II co-occurring with ES, providing a novel treatment alternative for clinical practice. Although the pathogenesis of MPS II combined with ES remains poorly understood, further research is required to investigate the intrinsic association between MPS and hematological diseases. Such investigations can advance the understanding of pathogenic mechanisms and provide a theoretical foundation for the development of more effective therapeutic strategies.

## Patient Perspective

The patient's families were satisfied with the diagnosis and treatment process.

## Data Availability

The original contributions presented in the study are included in the article/Supplementary Material, further inquiries can be directed to the corresponding author.
