# Supplementary material for: Merging evans syndrome with mucopolysaccharidosis type II: a case report
Source: Front Pediatr. 2026 May 4;14:1784387. doi: 10.3389/fped.2026.1784387 (PMC13180863; doi:10.3389/fped.2026.1784387)
Supplement: Supplementary file 1 [file Supplementaryfile1.docx]

**Merging Evans Syndrome with Mucopolysaccharidosis Type II: A Case Report**

Xinrui Wang^1^, Jing Zhang^1^, Yanhui Tang^3^, Chunyan Liu^1^, Peng Hu^2^, You Yang^1^, Hongying Chen^1,^*

^1^ *Department of Pediatrics, Children Hematological Oncology and Birth Defects Laboratory, The Affiliated Hospital of Southwest Medical University, Sichuan Clinical Research Center for Birth Defects, Luzhou, Sichuan, 646000, China*

^2^ *Department of Radiology, The Affiliated Hospital of Southwest Medical University, Luzhou, Sichuan, 646000, China*

^3^ *Department of Pediatrics Growth and Health Care, The Guangan People's Hospital, Guangan, Sichuan 638099, China*

*** Corresponding author details**

Hongying Chen: chen0040955@163.com

**Keywords:** Mucopolysaccharidosis Type II (MPS II); Evans Syndrome (ES); Iduronate-2-sulfatase (IDS); Hematopoietic Stem Cell Transplantation (HSCT); Case report

Figure S1


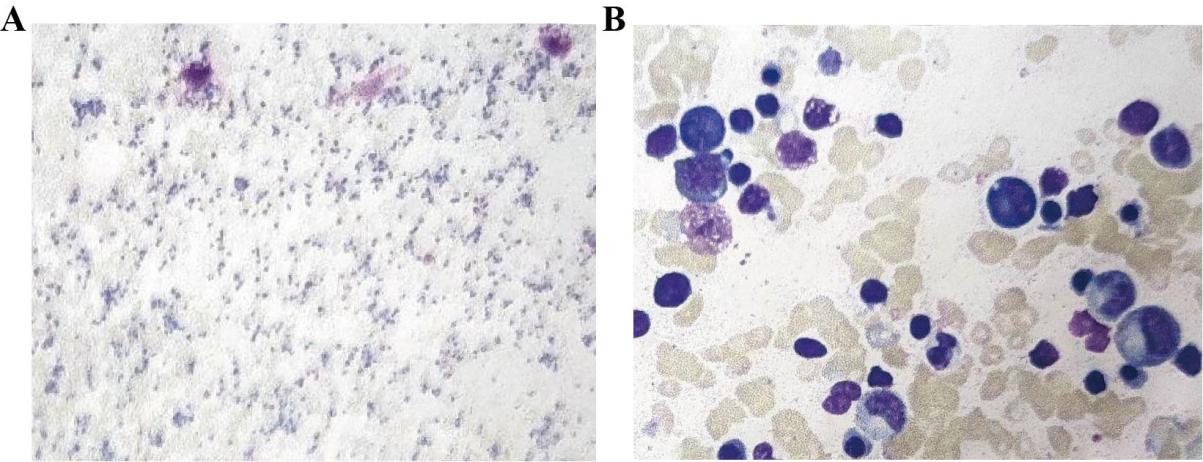


Figure S1. Wright's and Giemsa stain of Bone marrow cells (microscope)

1. Microscope (100×); (B) Microscope (400×); It reflected that the myelodysplasia was active.

Figure S2


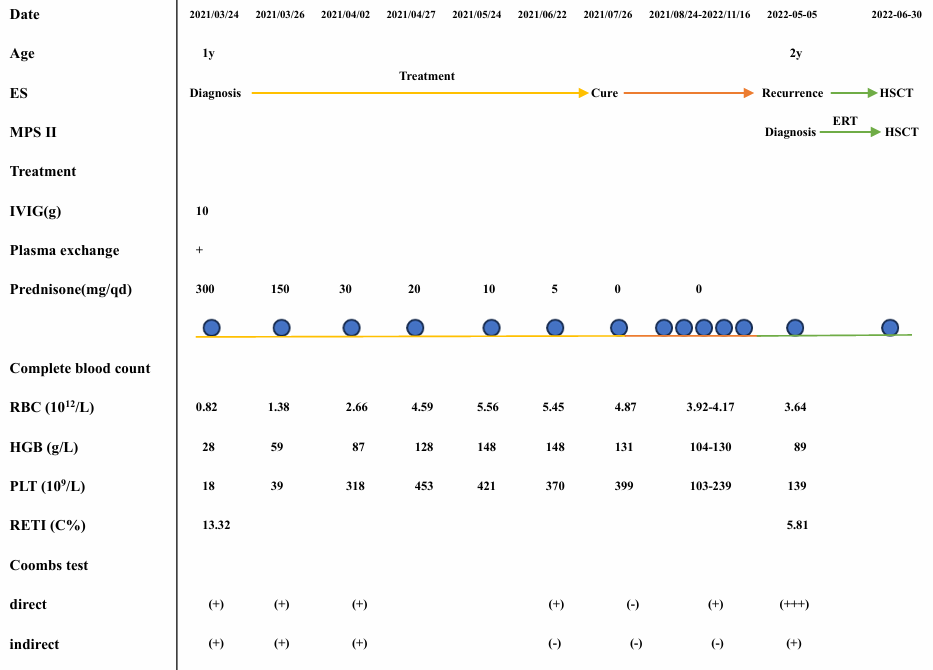


Figure S2. Treatment flow chart.

Figure S3


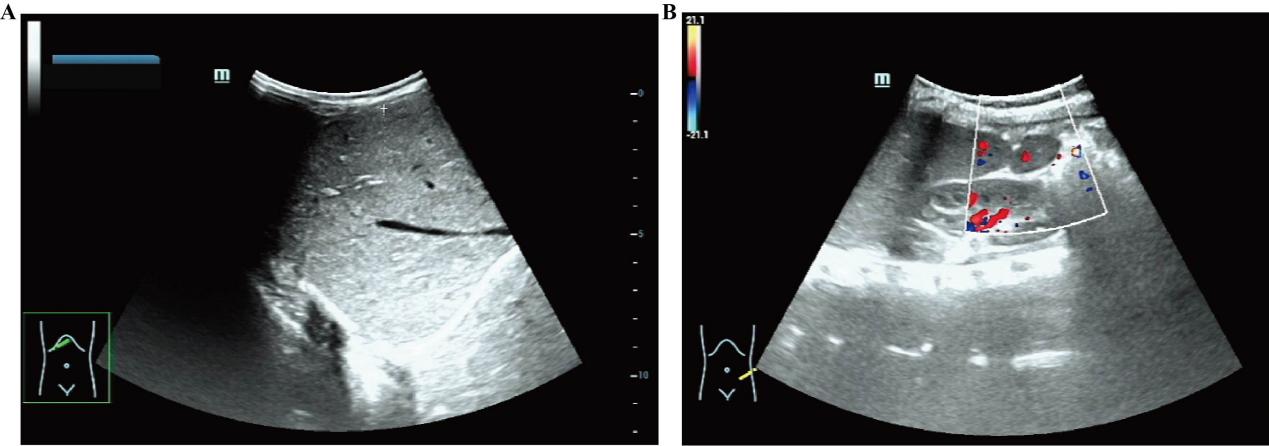


Figure S3. Abdominal ultrasound

1. Splenomegaly ；(B) Hepatomegaly.

Figure S4


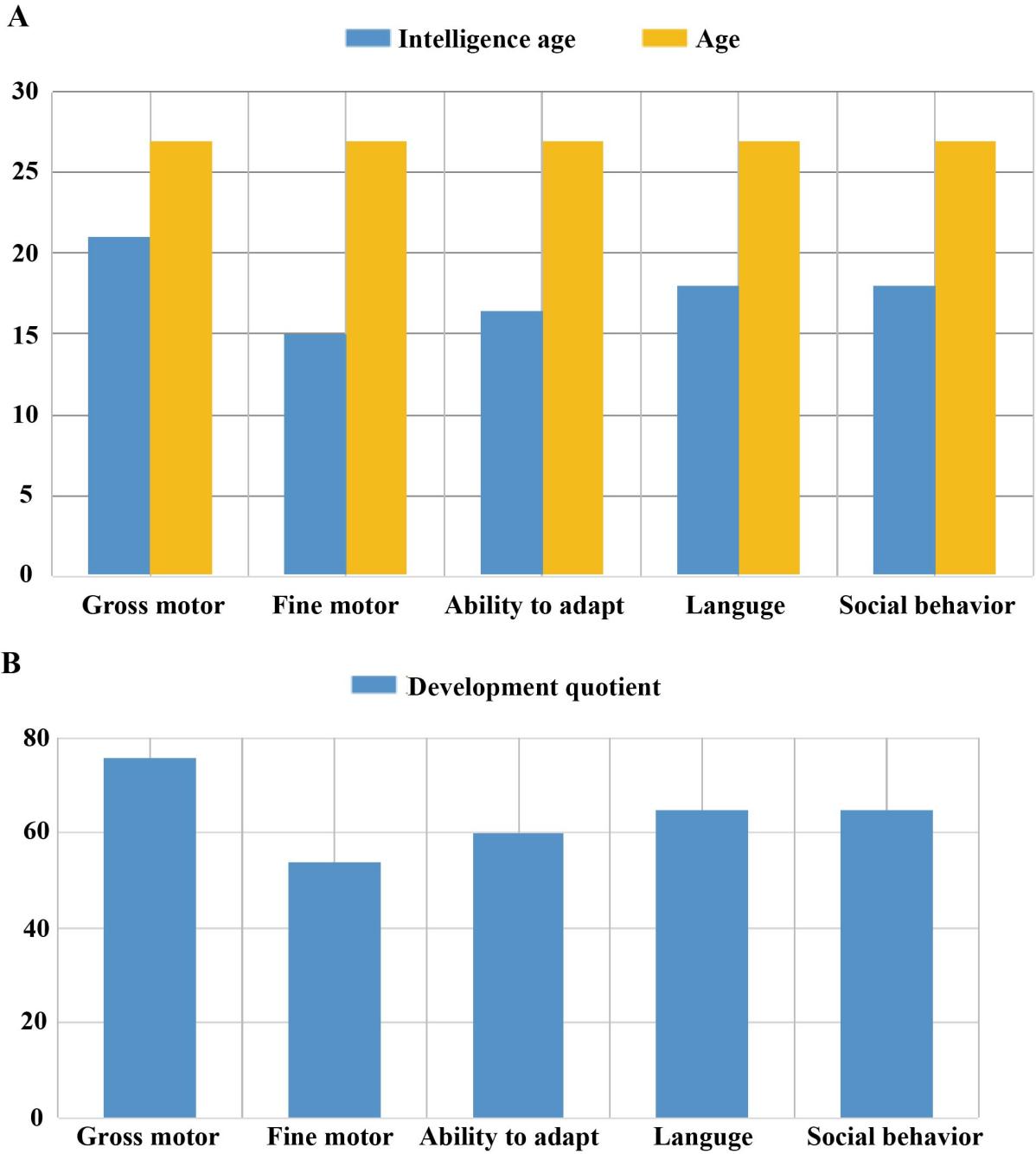


Figure S4. Intelligence assessment.

Figure S5


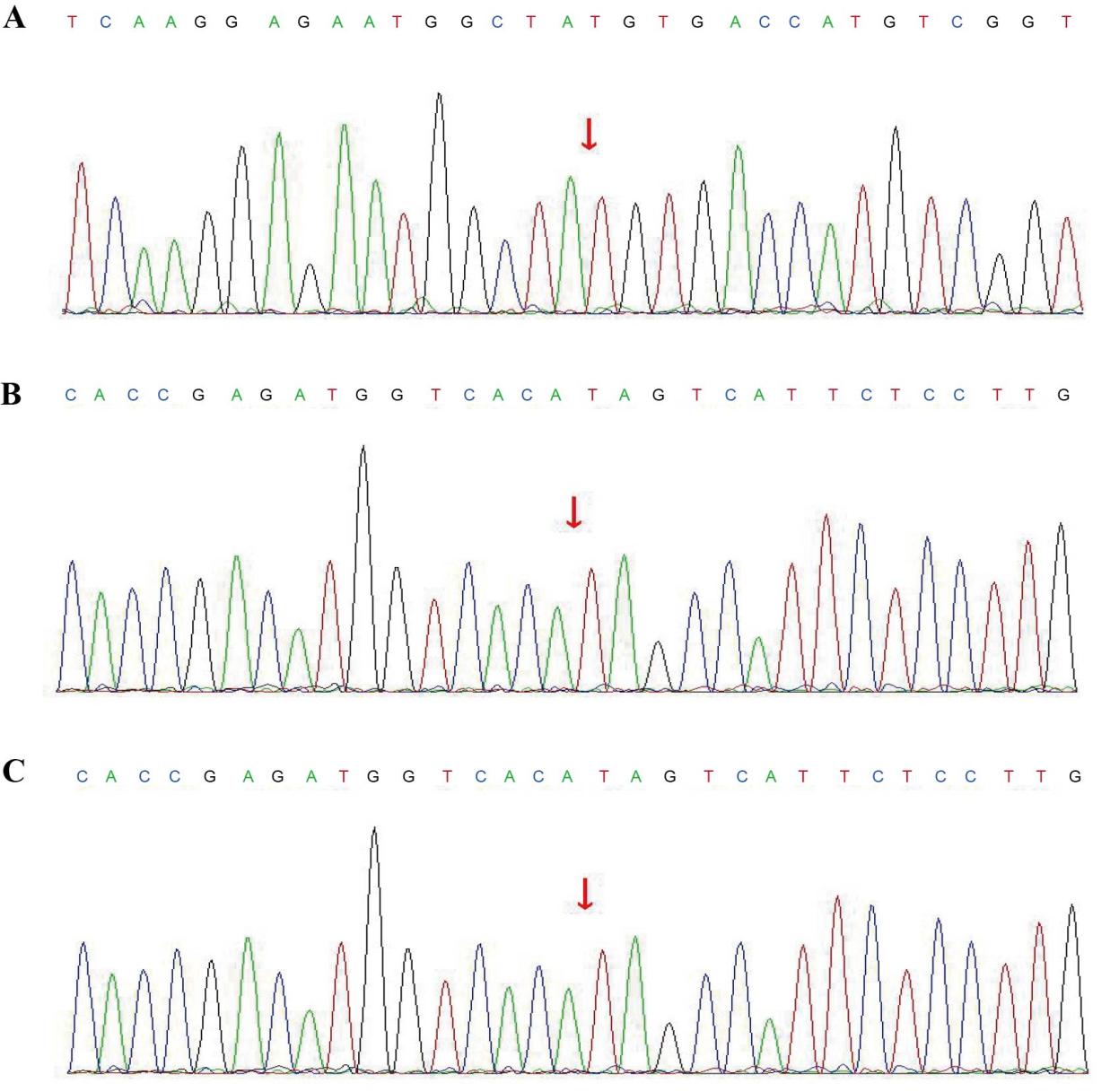


Figure S5. Sanger sequencing map of IDS gene

1. First sister; (B) Second sister; (C) Third sister; Sanger sequencing map of IDS gene showed Sisters had no IDS gene mutation.

Figure S6


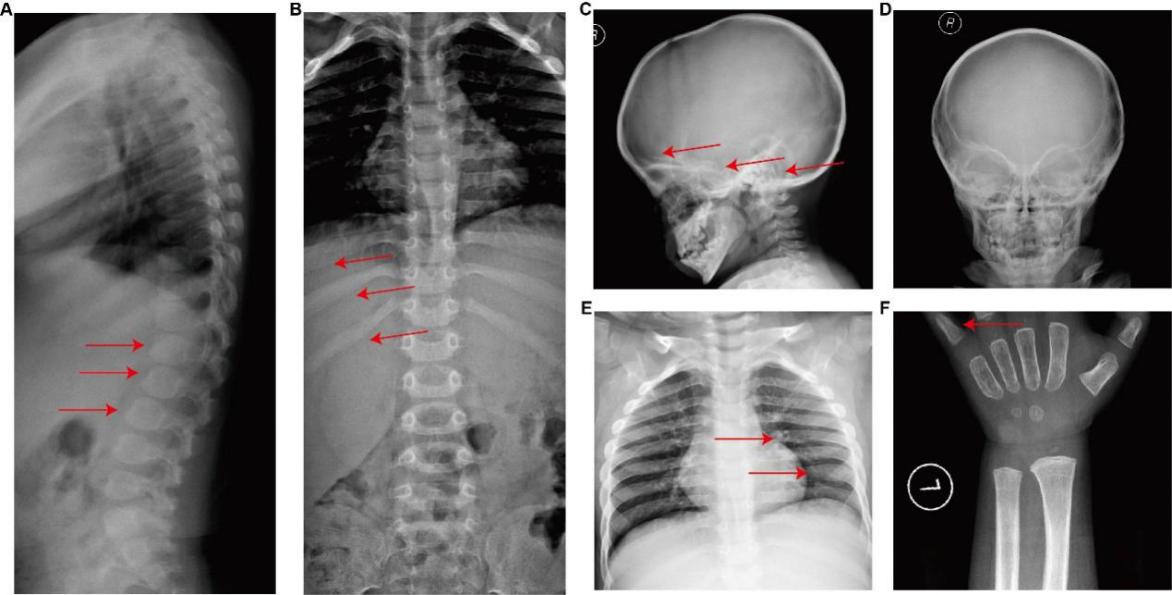


Figure S6. X-ray images of the patient

(A) Frontal image of the spine; (B) Lateral image of the spine; (C) Front view of the skull; (D) Lateral image of the skull; (E) Frontal image of the chest; (F) Frontal image of the wrist.

Figure S7


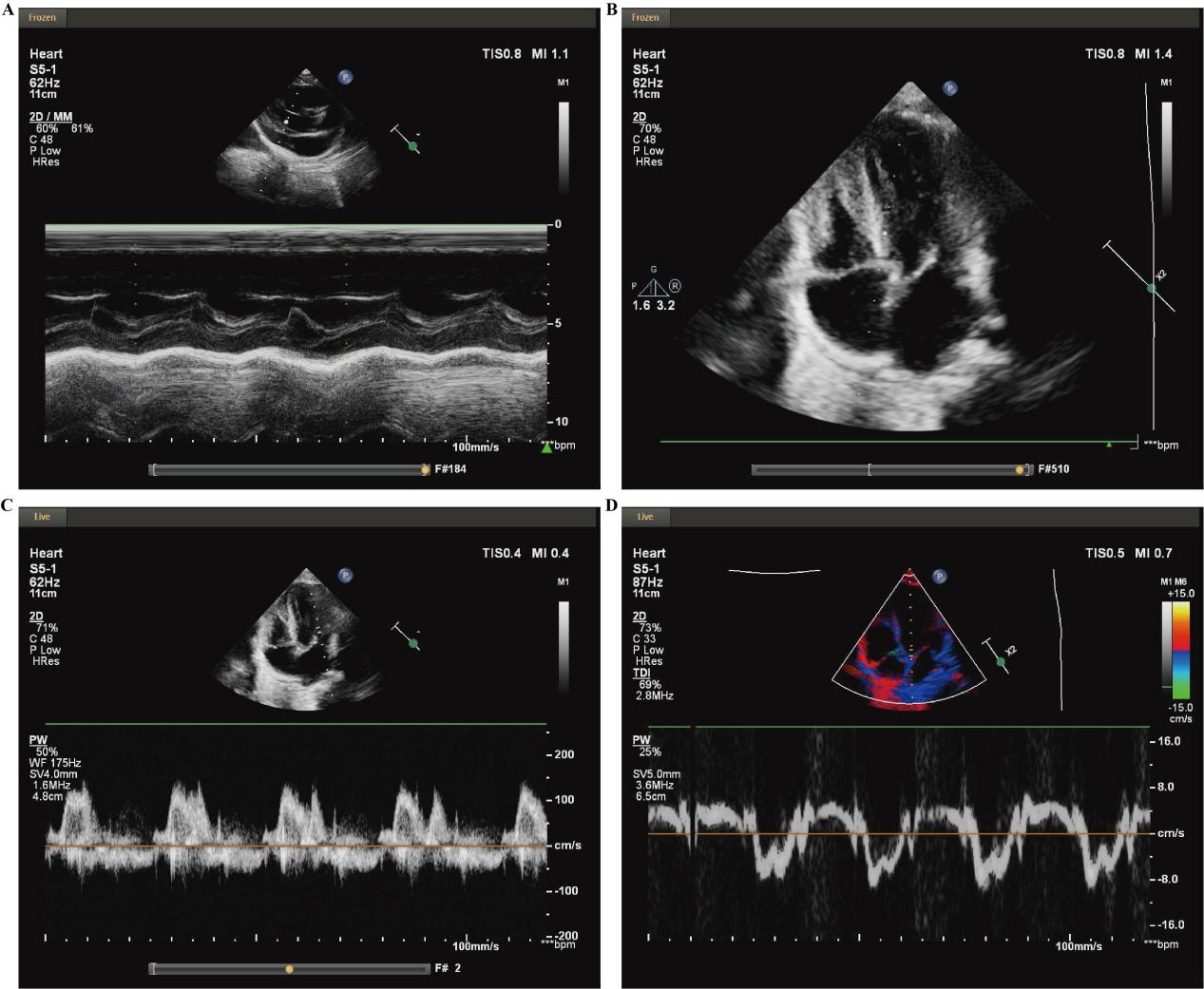


Figure S7. Echocardiography

1. Left ventricular M-mode echocardiography; (B) Apical four chamber section; (C) Mitral flow pattern; (D) Mitral annulus spectrum.
