# Supplementary material for: Merging evans syndrome with mucopolysaccharidosis type II: a case report
Source: Front Pediatr. 2026 May 4;14:1784387. doi: 10.3389/fped.2026.1784387 (PMC13180863; doi:10.3389/fped.2026.1784387)
Supplement: Supplementary file 2 [file Supplementaryfile2.docx]

**Merging Evans Syndrome with Mucopolysaccharidosis Type II: A Case Report**

Xinrui Wang^1^, Jing Zhang^1^, Yanhui Tang^3^, Chunyan Liu^1^, Peng Hu^2^, You Yang^1^, Hongying Chen^1,^*

^1^ *Department of Pediatrics, Children Hematological Oncology and Birth Defects Laboratory, The Affiliated Hospital of Southwest Medical University, Sichuan Clinical Research Center for Birth Defects, Luzhou, Sichuan, 646000, China*

^2^ *Department of Radiology, The Affiliated Hospital of Southwest Medical University, Luzhou, Sichuan, 646000, China*

^3^ *Department of Pediatrics Growth and Health Care, The Guangan People's Hospital, Guangan, Sichuan 638099, China*

*** Corresponding author details**

Hongying Chen: [chen0040955@163.com](mailto:chen0040955@163.com)

**Keywords:** Mucopolysaccharidosis Type II (MPS II); Evans Syndrome (ES); Iduronate-2-sulfatase (IDS); Hematopoietic Stem Cell Transplantation (HSCT); Case report

Table S1. High-Resolution HLA Typing of Recipient (2-Year-Old Male) and Donor (20-Year-Old Female Sibling)

| HLA | A | B | C | DRB1 | DQB1 | ABO/Rh |
| --- | --- | --- | --- | --- | --- | --- |
| Patient, 2y, M | 1101/1101 | 1502/4001 | 0702/0801 | 0403/1202 | 0301/0302 | A+ |
| Donor, 20y, F | 1101/1101 | 1502/4001 | 0702/0801 | 0403/1202 | 0301/0302 | A+ |

Note: The final stem cell product had a white blood cell count of 330.40 × 10^9/L, with neutrophil segments comprising 17%. A total of 43 mL of peripheral blood stem cells was transfused, resulting in a total mononuclear cell count of 8 × 10^8 cells/L/kg and a CD34+ cell count of 10.3 × 10^6 cells/L/kg, with CD34+ cells constituting 1.07% of the total.

Table S2. IDS Enzyme Activity Analysis

| Time after HSCT | pre-transplant | 3rd month | 6th month | 1st year | 1Y6M | 2nd year |
| --- | --- | --- | --- | --- | --- | --- |
| IDS (nmol/4hr/mg) | 0 | 45.06 | 35.34 | 50.74 | 48.97 | 64.98 |
| Reference |  | 19.89 | 12.60 | 25.44 | 35.40 | 14.57 |

Table S3. Organ Systems Involved in Case Reports of Mucopolysaccharidosis

| Organ or System Name | Number of cases |
| --- | --- |
| Fetus (including prenatal diagnosis) | 40 |
| Heart | 107 |
| Musculoskeletal | 100 |
| Nervous system | 129 |
| Eye | 130 |
| Digestive system | 12 |
| Ear, Nose, and Throat (ENT) | 17 |
| Teeth | 36 |
| Respiratory system | 76 |
| Immune system | 3 |
| Carpal Tunnel Syndrome | 21 |
| Hematologic System | 52 |
| Endocrine System | 5 |
| Urinary System | 9 |
| Umbilical Hernia | 3 |
| Diaphragmatic Hernia | 1 |
| Skin | 30 |
| Animal (exploring treatment, model establishment) | 29 |
| External Genitalia | 1 |
| Inguinal Hernia | 2 |
| Ascites | 2 |

Table S4. Case Reports on Hematological Aspects of Mucopolysaccharidosis

| Author | Disease | Animal Experiment | Gender | Age | Hematological manifestations | Treatment | Site | Follow-up |
| --- | --- | --- | --- | --- | --- | --- | --- | --- |
| J R Hobbs et al. | MPSI | - | Male | 1 year | - | Bone marrow transplantation (Graft-versus-host disease (GVHD) occurred) | - | Symptom improvement, Erythrocyte adenosine deaminase activity is normal. |
| C Messina et. al | MPSI | - | - | - | - | Bone marrow transplantation | - | - |
| R Miniero et. al | MPSII | - | - | - | - | Bone marrow transplantation | - | - |
| Majda Benedik-Dolnicar et. al | MPS I | - | - | - | Coexisting Hemophilia A | - | - | - |
| Sarar Mohamed | MPS III | - | Male | 4 years | Coexisting glucose-6-phosphate dehydrogenase deficiency and sickle cell/β+ thalassemia | - | - | - |
| C A Mullen | MPSII | - | Male | 10 months | Developed autoimmune hemolytic anemia 9 months after transplantation | unrelated umbilical cord blood transplantation | - | After 2 years, enzyme activity is normal, and growth and development are normal. |
| C Peters，W Krivit | MPSII | - | Male | - | - | unrelated umbilical cord blood transplantation | - | - |
| Burak Uz, et. al | MPSII | - | - | - | Coexisting newly diagnosed idiopathic thrombocytopenic purpura | - | - | - |
| K T Chen, et. al | MPSI | - | Female | 2 years | Complicated by acute myeloid leukemia (anemia, thrombocytopenia, and leukocytosis) | - | - | - |
| Cláudia Teixeira et. al | MPS VI | - | Male | 10 years | A large number of stained azureophilic granules (Alder–Reilly granules) found in peripheral blood leukocytes | - | - | - |
| P. Corti et. al | MPS I | - | - | - | Immune-mediated hemolytic anemia (IHA) occurred after hematopoietic stem cell transplantation (HSCT) | Hematopoietic stem cell transplantation with T-cell and B-cell depletion was performed. | - | - |
| J Kapelushnik et. al | MPS I | - | Male | 16 months | pre-transplant conditioning | Haploidentical peripheral blood stem cell transplant | - | 30 months post-transplant, clinical condition is good, counts are normal, no signs of graft-versus-host disease, no infections occurred, and neurological signs have stabilized. |
| Inusha Panigrahi et. al | MPS II | - | Male | 2 years | 1. MPS II complicated by persistent thrombocytopenia  2. Thrombocytopenia due to EBV infection  3 anemia, dyspnea, heart failure, hepatosplenomegaly, c  mitral valve prolapse with mitral regurgitation and severe left ventricular systolic dysfunction | 1. Platelet and red blood cell transfusions  2. Intravenous immunoglobulin transfusion  3. Oral prednisone | - | 2 years later, platelet count is normal, with contractures and knee valgus present. |
| Rahul Naithani | MPS I | - | Male | 16 months | 1. Pre-transplant conditioning  2. Complicated by cytomegalovirus infection and thrombocytopenia | 1. Haploidentical hematopoietic stem cell transplantation (HSCT)  2. Oral ganciclovir, platelet transfusions, and eltrombopag  3. Eltrombopag effectively increased platelet count to a level that no longer required platelet transfusions. | - | - |
| V Lee et. al | MPS VI | - | Male | 5 years | - | 1. Pre-transplant conditioning  2.Umbilical cord blood transplantation | - | 15 months post-transplant, N-acetylgalactosamine-4-sulfatase activity remains at normal levels. Clinical improvements noted in hepatosplenomegaly, facial and skin features, joint mobility, and resolution of purulent otitis media. He has returned to school and continues to perform well academically. |
| Akitaka Shibata et. al | MPS II | - | Male | 10 months | - | 1. Oral prednisone  2. Oral antihistamines | - | GAG accumulation persists after HSCT. |
| Jonas Alex Morales Saute et. al | MPS I | - | - | - | - | Hematopoietic stem cell transplantation | - | Successful hematopoietic stem cell transplant patients showed significant improvement on MRI after 3 years. |
| Simon Dulz et. al | MPS I | - | Male | 1.6 years | Severe bilateral scar-related eyelid entropion | Hematopoietic stem cell transplantation | - | Three months after surgical intervention, the patient showed sustained normal eyelid position. |
| S Meyer et. al | MPS II | - | Female | 8.5 years | - | Bone marrow transplantation from an MPS II patient | - | No clinical symptoms of mucopolysaccharidosis were observed 20 months after BMT. |
| Kenji Orii et. al | MPS VII | - | Female | 34 years | - | Hematopoietic stem cell transplantation (HSCT) at age 12 | p. Ala619Val | 22 years post-HSCT, β-glucuronidase activity in leukocytes was normal, and urine glycosaminoglycan excretion remained at normal levels. |
| [Rubal Jain](https://pubmed.ncbi.nlm.nih.gov/?term=Jain+R&cauthor_id=30983812) et. al | MPS IV | - | Female | 11 years | Preliminary blood test by Sysmex XN 1000 showed abnormal leukocyte scatter plot and signs of lymphocytosis. | - | - | - |
| Pankti Haria et. al | MPS VI | - | Female | 3 years | - | Received combined transplantation of umbilical cord blood (UCB) and bone marrow (BM) from a fully HLA-matched (6/6) donor (her sibling). | - | Follow-up 4 years post-transplant showed normal enzyme levels and no complications. Quality of life has improved. |
| Luis M Carbajal-Rodríguez et. al | MPS I | - | Male | Twins | - | ERT (started at 10 months) plus HSCT (started at 18 months) | Exon 6/IVS 6: c.767_793-15DUP112; Exon 8: c.1186_1188delCTG | Improvement in cardiovascular symptoms and psychomotor development |
| Anneliese L Barth et. al | MPS II | - | Male | Fetal | Positive family history of severe MPS forms. | Received umbilical cord blood hematopoietic stem cell transplantation at 70 days old. | Familial p.Arg88His mutation (p.R88H) on Exon 3. | At age 7, there were minor signs of multiple skeletal dysplasia and hearing loss. Growth charts were normal. |
| Srividya Sreekantam et. al | MPS II | - | Male | Sibling A: Diagnosed with Hunter syndrome at age 2.  Sibling B: Diagnosed shortly after birth. | - | Sibling A: Intravenous and intrathecal  Sibling B: Early HSCT at 6 weeks old. | Both siblings confirmed as homozygous for the pathogenic mutation G224E in IDS. | The scale score of sibling B is higher than that of sibling A. |
| [Stefano Giardino](https://pubmed.ncbi.nlm.nih.gov/?term=Giardino+S&cauthor_id=33314581) et. al | MPS I | - | - | - | - | Allogeneic hematopoietic stem cell transplantation | - | - |
| L Peterson et. al | MPS VII | - | - | Infant | Reported morphological, cytochemical, and ultrastructural pathology of blood and bone marrow in an infant with type VII mucopolysaccharidosis (MPS VII). | - | - | - |
| Jing Chen et. al | MPS | - | MPS I and MPS VI | - | - | Peripheral stem cells were collected from a 9/10 high-resolution matched unrelated donor and a matched sibling carrier donor. | - | MPS I has been followed for 25 months, and MPS VI for 28 months, with patients generally doing well. |
| Albina Tummolo et. al | MPSI | - | - | Female | Skeletal changes | underwent two hematopoietic stem cell transplants. | - | - |
| J Nishioka et. al | MPSI | - | - | - | Joint Stiffness | Comparison of the treatment effect  s of leukocyte transfusion (LT) versus plasma infusion (PI). | - | - |
| Éliane Beauregard-Lacroix et. al | MPSIII | - | 16 years | Female | Moderate pancytopenia. | - | Two variants in NAGLU (OMIM: 609701): c.2135del (p.[Lys712Serfs*95]) and a 7.31 Kb deletion on 17q21.2 chromosome encompassing exons 1–5 of NAGLU. | - |
| H Endo et. al | MPS | - | 22 years | Male | The patient presents with hereditary thrombocytopathy and color blindness. | - | - | - |
| Chester B Whitley | MPS VI | - | 22 years | Male | - | He received an allogeneic bone marrow transplant from an HLA-matched sibling donor. | - | Maintaining complete remission 20 years post-transplant. |
| G Remérand et. al | MPSI | - | 4 years | Female | - | Underwent allogeneic bone marrow transplantation. | Two mutations: p.P533R and p.E178K. | Five years post-BMT, there was an improvement in clinical overload, and enzyme activity returned to normal. |
| J J Ortega Aramburu et. al | MPSI | - | 9 years | Female | - | Underwent hematopoietic stem cell transplantation from her mother. | - | At 12 months, glycosaminoglycan (GAG) levels were normal, and Clinical features improved. |
| Francesca Furlan et. al | MPSVII | - | 8 months | Male | - | Successfully underwent hematopoietic cell transplant (HCT) at 14 months of age. | Molecular analysis showed homozygous variant c.1617C > T, resulting in synonymous mutation p.Ser539=. | The patient died from respiratory failure due to respiratory syncytial virus infection at 25 months of age. |
| Young Bae Sohn et. al | MPSVI | - | 15 years | Female | - | Underwent hematopoietic stem cell transplantation from an HLA-matched sibling donor (sister) at age 5. | - | Enzyme replacement therapy after hematopoietic stem cell transplantation improved joint mobility. |
| J J Hopwood et. al | MPSI | - | 12 years and 14 years | - | - | Underwent hematopoietic stem cell transplantation. | Homozygous mutation W402X. | BMT significantly slowed the clinical regression of the W402X phenotype. |
| Murat Doğan et. al | MPS VI | - | - | - | - | Enzyme replacement therapy. | - | - |
| Ching-Chia Wang et al | MPS VI | - | 10 years | Female | - | Bone marrow from her HLA-matched brother. | - | Within 12 years post-BMT, motor function improved, and infections occurred rarely. |
| F Alvaro et. al | MPSVI | - | - | - | - | Allogeneic bone marrow transplantation. | - | - |
| C Navarro et. al | MPSI | - | 11 years | Female | - | Underwent bone marrow transplantation. | - | Fibroblast morphology of connective tissue is nearly normal 21 months post-BMT. |
| Luisa Sisinni et. al | MPSVII | - | 2 years | Female | Skeletal issues, neurocognitive changes | Underwent bone marrow transplantation. | - | Achieved stable complete donor engraftment and normal enzyme levels during 6 years of follow-up. |
| Mohammad Reza Alaei et. al | MPSIII | - | 4 years | Male | - | - | NAGLU：c.625A>C:(p.Thr209Pro）  GCDH：c.1298C>T:(p.Ala433Val） | - |
| S Yatziv et. al | MPSII | - | - | - | - | Long-term infusion of fresh plasma. | - | In the first six months of treatment, the clinical status of the siblings was characterized by accelerated growth. |
| Patricia Dubot et. al | MPSVII | - | 2 weeks | Male | - | ERT started at 4 months of age, followed by hematopoietic stem cell transplantation (HSCT) at 13 months. ERT was discontinued 6 months after HSCT. | Three missense mutations: c.422A>C, c.424C>T, and c.526C>T. | At age 4, demonstrated normal psychomotor development, stable growth curve, with no hepatosplenomegaly or other organ involvement. |
| Narutoshi Yamazaki et. al | MPSI | - | 18 days | Female | - | HSCT was performed at 9 months old. | - | Mild multiple bone exostosis and mild signs of valvular heart disease. |
| R Annibali et. al | MPSII | - | - | - | - | Hematopoietic stem cell transplantation. | - | Changes in white matter remained stable over time. |
| Erica Corda et. al | MPSVII | Animal Experiment | - | - | - | - | GAG exposure within lysosomal-like primary granules of lysosomes or granulocyte precursors. | - |
| H Pande et. al | MPSIII | - | - | Female | Associated with thrombocytopenic purpura. | - | - | - |
